# Social media addiction, maternal mental health, and children’s oral health behaviours among immigrants

**DOI:** 10.1371/journal.pone.0357008

**Published:** 2026-08-27

**Authors:** Solmaz Bohlouli, Devarasa Murugeshappa, Babak Bohlouli, Maryam Amin

**Affiliations:** 1 Department of Medicine, University of Alberta, Edmonton, Canada; 2 Department of Dentistry, University of Alberta, Edmonton, Canada; 3 Assistant Adjunct Professor, Department of Dentistry, University of Alberta, Edmonton, Canada; 4 Professor and Associate Chair of Research Mike Petryk, School of Dentistry, University of Alberta, Edmonton, Canada; University of the Witwatersrand Johannesburg Faculty of Health Sciences, SOUTH AFRICA

## Abstract

**Objectives:**

Oral health disparities persist among immigrants. Little is known about how social media shapes the socio-psychological environment in which parenting and health behaviours occur. This study examined the link between immigrant mothers’ anxiety and depression and children’s oral health behaviours (OHBs) and evaluated social media addiction as a mediator or moderator.

**Methods:**

This cross-sectional study recruited immigrant parents of children aged 2–12 years in Edmonton, Canada. Parents completed a questionnaire assessing demographics, children’s OHBs (dental visits, toothbrushing, diet), anxiety and depression (The Hospital Anxiety and Depression Scale, HADS-14; scored 0–21), and social media addiction (Bergen-6; scored 6–30). Logistic regression, mediation, and moderation analyses were performed.

**Results:**

302 questionnaires were analyzed. Mean (SD) ages of mothers and children were 37.7 (6.8) and 6.7 (3.3) years, respectively; 49% of children were female, and 65% born in Canada. On average, parents had lived in Canada for 9.9 years, and 68% had higher education. Higher maternal anxiety reduced the odds of child toothbrushing by 14% (OR: 0.86, 95% CI: 0.74 0.98), and higher depression was associated with a 21% reduction (OR: 0.79, 95% CI: 0.63–0.93). Each one-unit increase in anxiety and depression corresponded to a 49% and 51% increase in social media addiction scores (p < .05). No mediation or moderation effects were found.

**Conclusions:**

Maternal anxiety and depression reduced children’s toothbrushing, with no mediation by social media addiction. Integrating mental health screening into dental care can improve oral health in immigrant communities, fostering better outcomes and inclusive policies.

## Introduction

Optimal oral health in children is essential for their overall well-being, as it influences their ability to eat, speak, and socialize comfortably [1]. Early prevention of oral health issues, such as dental caries, is critical, as these conditions can lead to lifelong consequences [1]. Globally, dental caries is the most prevalent yet preventable chronic disease, affecting the majority (60–90%) of school-aged children [2,3]. In Canada, dental-related problems alone have significant public health implications, accounting for over 2.26 million missed school days annually and representing one-third of day surgeries for children aged 1–5 years [2]. Oral health conditions vary across age groups, showing an increase in chronic mouth pain and untreated caries from early childhood to older ages. Although most individuals brush their teeth twice daily, regular brushing is less common among young children, and consistent flossing remains uncommon [3]. These reports highlight the importance of promoting preventive oral health practices throughout childhood, emphasizing that establishing good oral health habits early in life is crucial for long-term well-being.

Parents play a pivotal role in fostering good oral health for their children [1,4]. Key aspects of this involvement include establishing oral hygiene routines, encouraging healthy dietary habits, and seeking routine dental care. Research has shown that parents who actively monitor children’s oral health behaviour contribute significantly to better oral health outcomes [1]. Additionally, parental oral health literacy, knowledge, and attitudes greatly influence children’s behaviours and access to dental services, which are critical in preventing oral diseases and conditions such as early childhood caries (ECC) [1,4]. However, access to preventive care and education can be limited for some families.

Immigrant families encounter numerous systemic and cultural barriers, including geographic, economic, and linguistic challenges, alongside difficulties navigating the healthcare system, which delay access to essential care [4,5]. These obstacles gradually undermine the “healthy immigrant effect,” a phenomenon in which newly arrived immigrants often demonstrate better health outcomes than the native-born population, as limited access to primary and preventive healthcare services adversely affects children’s oral health [5]. Furthermore, factors such as competing household responsibilities and a lack of oral health literacy contribute to the prevalence of untreated dental issues in children [4]. First-generation immigrant children in Canada face disproportionately higher risks of poor oral health outcomes compared to their Canadian-born peers [4,6–9]. These stressors may also create conditions that heighten vulnerability to socio-digital pressures, including reliance on social media for support and information.

Adopting a socio-ecological perspective that accounts for digitally mediated environments, this study views contemporary parenting as unfolding within a digitally saturated space. Social media can thus be understood not merely as a communication tool but as a socio-technical system embedded within a broader socio-digital ecology that shapes social interactions, emotional well-being, and daily caregiving practices. For immigrant families in particular, networked platforms such as Facebook, WhatsApp, WeChat, and YouTube serve as key infrastructures for maintaining transnational ties, exchanging parenting information, seeking community support, and navigating the acculturation process. These platforms shape not only communication patterns but also emotional well-being, access to information, and daily caregiving practices.

Social media significantly influences immigrants by offering platforms to maintain connections with their homeland while integrating into a new society [10]. These platforms support cultural adaptation by providing resources and reducing stress, but over-reliance can lead to addiction, reinforcing cultural separation and dependency [10]. Immigrants often use social media more than non-immigrants to bridge cultural gaps and manage loneliness. Excessive screen time can negatively affect parenting and child outcomes [11]. The concept of *technoference*, defined as everyday interruptions in interpersonal interactions due to digital device use, was introduced by McDaniel and Radesky (2018) [12]. In the context of parent–child relationships, technoference can disrupt engagement by reducing responsiveness and quality time, potentially contributing to behavioral problems, emotional difficulties, and feelings of neglect in children (13). Furthermore, excessive social media use can heighten parental stress and feelings of inadequacy [13]. Parents’ social media addiction can indirectly affect children’s oral health by exacerbating parental depression and anxiety, which are linked to impaired emotional regulation, loneliness, diminished self-esteem, and strained relationships [13, 14]. These mental health challenges can hinder parents’ ability to manage their children’s oral health, including arranging dental visits and fostering consistent oral health practices, ultimately affecting caries experience [14–22].

Extending this body of research, social media can be understood not merely as a communication tool but as a socio-technical system embedded in immigrant settlement, cultural negotiation, and digitally mediated parenting. Problematic or addictive social media use may shape the emotional climate of the household and influence parental capacity to support consistent health-promoting routines such as toothbrushing.

**Fig 1:**
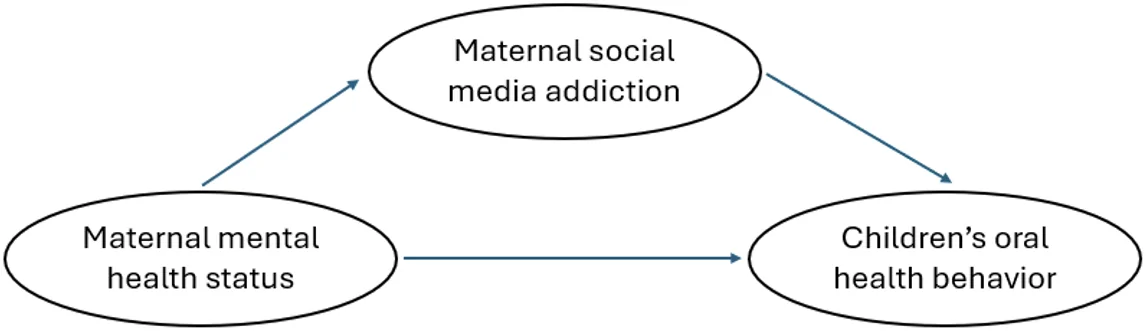
Hypothesized pathways linking maternal mental health and social media addiction to children’s oral health behavior.

These findings highlight the importance of exploring the potential link between excessive social media use among immigrant parents and adverse oral health outcomes in their children. This relationship may be further influenced by parental mental health challenges, such as depression and anxiety, emphasizing the importance of integrating mental health support into oral health education and intervention programs. However, the specific impact of parental social media use on children’s oral health within immigrant populations, particularly considering the potential mediating role of parental mental health, remains understudied. Therefore, this study examines these relationships within a broader socio-digital context by investigating: (1) whether maternal mental health is associated with children’s oral health behaviours, and (2) whether the association between maternal mental health and children’s oral health behaviours is mediated or moderated by social media addiction. Fig 1 visualizes the hypothesized associations for the study’s conceptual framework.

## Methods

A cross-sectional observational study was conducted between 2021 and 2024 in Edmonton, Alberta, Canada. Ethical approval was obtained from the Health Research Ethics Board at the University of Alberta (Pro00112790). Written informed consent was obtained from all participants before data collection.

The study sample comprised immigrant mother–child dyads with children aged 2–12 years. Recruitment targeted diverse ethnic communities, including South Asian, Middle Eastern, Chinese, Filipino, and African populations, with a starting date of 01/09/2021, ending date of 10/08/2024. Community engagement and data collection were facilitated through the Multicultural Health Brokers Cooperative. Because social media plays an important role in communication within many immigrant communities, outreach efforts were designed to align with these socio-digital communication patterns and relied on culturally grounded, community-based approaches. To ensure inclusivity, mothers with limited English proficiency received support from bilingual community health brokers or interpreters, enabling them to complete the survey in a culturally sensitive and linguistically appropriate manner.

Mothers completed a structured four-part questionnaire that collected information on:socio-demographic characteristics, children’s oral health behaviours, maternal anxiety and depression, and maternal social media addiction. Demographic and contextual factors included maternal age, maternal education level, household income, number of children, child’s age, child’s birthplace (Canada or abroad), and maternal length of residence in Canada. The independent variables included Maternal Mental Health, assessed by using the Hospital Anxiety and Depression Scale (HADS), which consists of 14 items (seven each for anxiety and depression). Scores for each subscale range from 0 to 21, with higher scores indicating greater symptom severity. The HADS is a validated screening tool and does not provide clinical diagnoses of anxiety and depression. Social Media Addiction (SMA) was measured by using the Bergen Social Media Addiction Scale (BSMAS), a six-item instrument with scores ranging from 6 to 30, where higher scores reflect greater levels of problematic social media use. Dependent Variables included children’s oral health behaviours (OHBs), evaluated through parent-reported measures, including brushing frequency, frequency of sugary food consumption, dental visits in the past year, and mothers’ reported assessment of their child’s oral health status.

All analyses were conducted using SAS version 9.4 (SAS Institute Inc., Cary, NC). Descriptive statistics were used to characterize the study sample. Continuous variables were summarized using mean and standard deviation (SD), median and interquartile range (IQR), or minimum and maximum values, as appropriate. Categorical variables were reported as frequencies and percentages. Inferential analyses included multivariate logistic regression to examine associations between maternal mental health, social media addiction, and children’s oral health behaviours. Moderation and mediation analyses were conducted to test whether social media addiction indirectly influenced the relationship between maternal mental health and oral health behaviours. Statistical significance was determined using a 95% confidence interval (CI) and a two-sided p-value < 0.05.

Causal mediation analysis was conducted to quantify the extent to which the association between maternal mental health, anxiety and depression, and the children’s oral health behavior was mediated through social media use. The total effect of anxiety and depression on the oral health behaviour was decomposed into the natural direct effect (NDE) and natural indirect effect (NIE) via social media use within a counterfactual framework. Two regression models were specified: (1) a mediator model estimating the association between anxiety and social media use, and (2) an outcome model including both anxiety and social media use as predictors of the outcome. Both models were adjusted for covariates, including the mother’s age, child’s age, sex of child (male or female), number of children in care, whether the child was born in Canada, dental coverage for children, and the mother’s education level. For binary outcomes and mediator variables, logistic regression models were specified. The proportion mediated was calculated to quantify the indirect pathway’s relative contribution.

## Results

A total of 302 immigrant mothers of children aged 2–12 years participated in this study. The mean (SD) ages of mothers and children were 37.7 (6.8) and 6.7 (3.3) years, respectively. Most mothers (68%) had attained a post-secondary level of education and had lived in Canada for an average of 9.9 years. Sixty-five percent of children were born in Canada. Detailed demographic characteristics are presented in Table 1.

**Table 1:** Socio-Demographic Characteristics of Participants (N = 302).

Characteristics	N (%) or Mean (SD)
**Cohort size**	302 (100%
**Child Characteristics**	
Age, years	6.75 (3.09)
Gender (female)	147 (48.7)
Born in Canada	195 (64.6)
Living with both parents	281 (93.0)
Number of children in care	1.97 (0.79)
**Maternal Characteristics**	
Age, years	37.66 (6.74)
Years residing in Canada	9.8 (5.23)
Immigration category	
Economic immigrant (Federal Skilled Worker/Canadian Experience Class)	165 (54.6)
Family sponsorship	80 (26.5)
Humanitarian/refugee/student/other	57 (18.9)
Citizenship status	
Canadian citizen	113 (37.4)
Permanent resident	146 (48.3)
Other status	43 (14.3)
Marital status (married/common-law)	280 (92.7)
Education (college or higher)	269 (89.1)
**Household Characteristics**	
Monthly income	
<$2,000	49 (16.2)
$2,000–$4,000	87 (28.8)
> $4,000	110 (36.4)
Dental coverage for child	202 (66.9)
Private	170 (56.3)
Public/government	32 (10.6)
Other/unknown	14 (4.6)

### Children’s oral health behaviours

About 61.9% of children had visited a dentist in the past year; among them, 80.7% attended for regular check-ups. More than half of the children (58.1%) brushed their teeth at least twice daily, and 64.2% consumed sugary foods less than once a day. However, only 48% of mothers rated their child’s oral health as excellent or very good. Detailed oral health behaviours are presented in Table 2.

**Table 2:** Children’s Oral Health Practices, Dental Visits, and Perceived Oral Health Status.

Oral Health Outcomes	Category	N (%)
Oral Health Practices	Daily tooth brushing frequency	
	Once or less than once per day	127 (42.0)
	Twice or more per day	175 (58.0)
	Age child began brushing independently	
	≤ 2 years	121 (40.2)
	3–5 years	92 (30.6)
	≥ 6 years	49 (16.3)
	Does not brush independently	39 (13.0)
	Sugary food and drink intake frequency	
	Never or less than daily	194 (64.2)
	Once daily or more often	107 (35.4)
Dental Visits	Child’s last dental examination	
	Within the last 12 months	187 (61.9)
	Over one year ago or never	115 (38.1)
	Reasons for dental visit	
	Regular check-up or non-urgent	244 (80.7)
	Urgent or other	24 (7.9)
Children’s Oral Health	Mothers’ reported oral health status	
	Excellent or very good	145 (48.0)
	Good	105 (34.8)
	Fair or poor	52 (17.2)
	Parent-reported dental caries status	
	Yes	112 (37.1)
	No	143 (47.4)
	Not sure	47 (15.6)

### Maternal mental health and child oral health behaviours

The mean maternal anxiety and depression scores were 7.5 (SD = 4.2) and 6.6 (SD = 3.4), respectively, and were strongly correlated (β = 0.61, p < 0.0001). Higher maternal anxiety was significantly associated with younger maternal age (β = –0.16, p < 0.0001) and lower household income (β = –0.19, p < 0.001). Mothers without Canadian citizenship also reported significantly higher anxiety levels compared to Canadian citizens. Multivariable logistic regression demonstrated that both higher maternal anxiety and higher maternal depression scores were associated with reduced odds of children brushing their teeth at least twice daily (Anxiety: OR = 0.86, 95% CI: 0.74–0.98; Depression: OR = 0.79, 95% CI: 0.63–0.93), Table 3. In contrast, neither maternal anxiety nor depression was significantly associated with children’s sugar intake (Anxiety: OR = 1.00, 95% CI: 0.94–1.06; Depression: OR = 1.00, 95% CI: 0.94–1.06) or with frequency of dental visits (Anxiety: OR = 1.01, 95% CI: 0.96–1.08; Depression: OR = 1.01, 95% CI: 0.96–1.08).

**Table 3:** Regression results for toothbrushing children’ oral health behaviors.

**Effect**	**Point Estimate**	**95% Wald Lower Limit**	**95% Wald Upper Limit**
**Anxiety score**	**0.86**	**0.74**	**0.98**
Sex (Male vs female)	0.45	0.12	1.73
Dental insurance	0.40	0.11	1.49
Mother’ age	1.00	0.90	1.11
Child’s age	1.13	0.91	1.41
Number of children in care	0.73	0.37	1.43
Child born in Canada (Yes vs no)	0.81	0.48	1.37
Maternal Education (over high school vs less than high school	1.18	0.57	2.45
**Depression score**	**0.79**	**0.63**	**0.93**
Sex (Male vs female)	2.14	0.56	8.16
Dental insurance (Yes vs no)	0.49	0.13	1.85
Mother’ age	1.01	0.92	1.10
Child’s age	1.10	0.89	1.35
Number of children in care	0.78	0.39	1.55
Child born in Canada (Yes vs no)	0.83	0.50	1.38
Maternal Education (over high school vs less than high school	1.26	0.59	2.71

### Maternal mental health and social media addiction

The mean maternal social media addiction (SMA) score was 15.9 (SD = 5.1). Higher levels of maternal anxiety (β = 0.41, p < 0.05) and depression (β = 0.31, p < 0.05) were significantly associated with greater SMA scores. SMA scores decreased with increasing maternal age (β = –0.17, p < 0.05) and with higher household income (β = –0.21, p < 0.05).

Based on the analyses conducted, maternal mental health was associated only with children’s toothbrushing behaviour. Subsequent analyses were undertaken to examine the potential mediating effect of social media addiction on this association. Depression was significantly associated with toothbrushing behavior (total effect: OR = 0.79, 95% CI: 0.63–0.93). Mediation analysis demonstrated a statistically significant natural direct effect (OR = 0.80, 95% CI: 0.65–0.95), indicating that depression exerts an independent influence on the outcome. In contrast, the natural indirect effect mediated through social media use was not statistically significant (OR = 0.98, 95% CI: 0.93–1.03), suggesting no evidence of mediation via increased social media use, Table 4.

**Table 4:** Direct, indirect, and total effects of maternal mental health on children’s tooth brushing behavior.

Depression	Brushing			
	Estimates	95% CI		P-value
Odds Ratio Total Effect	0.78	0.63	0.93	0.00
Odds Ratio Natural Direct Effect (NDE)	0.80	0.65	0.95	0.01
Odds Ratio Natural Indirect Effect (NIE)	0.98	0.92	1.03	0.44
Percentage Mediated	8.16	−12.90	29.22	0.45
Percentage Due to Interaction	1.14	−7.12	9.40	0.79
Percentage Eliminated	11.35	−21.85	44.56	0.50
	
**Anxiety**	**Brushing**			
	Estimates	95% CI		P-value
Odds Ratio Total Effect	0.86	0.74	0.98	0.03
Odds Ratio Natural Direct Effect (NDE)	0.87	0.74	1.01	0.07
Odds Ratio Natural Indirect Effect (NIE)	0.99	0.93	1.04	0.65
Percentage Mediated	8.47	−29.54	46.47	0.66
Percentage Due to Interaction	0.18	−4.11	4.47	0.93
Percentage Eliminated	9.87	−36.70	56.44	0.68

Similarly, anxiety was significantly associated with toothbrushing behavior (total effect: OR = 0.86, 95% CI: 0.74–0.98). The natural direct effect was of borderline statistical significance (OR = 0.87, 95% CI: 0.74–1.01), indicating a predominantly direct influence of anxiety on the outcome. The natural indirect effect through social media use was not statistically significant (OR = 0.98, 95% CI: 0.93–1.03), further supporting the absence of a mediating role for social media use in this association (Table 4).

Moderation and mediation analyses showed that SMA did not significantly alter the relationship between maternal mental health and children’s oral health behaviours (p > 0.05). The alternative pathway, with SMA as the independent variable and maternal mental health as the mediator, was not examined as it was beyond the scope of the pre-registered analysis plan. These findings suggest that while social media addiction and maternal mental health are closely linked within immigrant mothers’ socio-digital environments, their influence on children’s oral health behaviours appears to operate through distinct pathways rather than through reciprocal mediation.

## Discussion

The present study examined the associations between maternal mental health, social media addiction (SMA), and children’s oral health behaviours (OHBs) among immigrant mother–child dyads in Edmonton, Canada. It explored whether SMA mediated or moderated the relationship between maternal psychological distress and children’s OHBs. The findings revealed that higher maternal anxiety and depression were significantly associated with lower odds of children brushing their teeth twice daily. At the same time, no significant associations were found with children’s sugar intake or dental visits. By analyzing these patterns within a digitally saturated context, this study highlights how maternal psychological well-being and problematic social media use coexist within the socio-digital environments of immigrant families, shaping, but not fully determining, daily caregiving routines.

The negative association between maternal mental health challenges and children’s toothbrushing practices aligns with existing research demonstrating that maternal anxiety and depression can disrupt daily caregiving tasks, including hygiene routines [23]. These mental health conditions may reduce maternal capacity to maintain consistent schedules, monitor children’s behaviours, and provide the emotional support necessary for routine health behaviours such as toothbrushing. Within a broader social media context, these disruptions may be intensified by the pressures of constant connectivity, digital comparison cultures, and the emotional demands associated with transnational social networks maintained through social media platforms. Such socio-digital pressures may contribute to psychological overload, thereby reducing parents’ capacity to engage in consistent health-promoting routines.

In contrast, maternal anxiety and depression were not associated with children’s sugar intake or dental visits. These behaviours may be less directly influenced by maternal emotional availability and more shaped by structural, financial, or systemic factors such as access to dental insurance, cost of services, and transportation. Access to public dental programs, community clinics, and culturally tailored outreach services may facilitate dental attendance among immigrant families even when maternal psychological distress is present [24]. In Edmonton, community-based oral health initiatives and culturally responsive programs offered by Alberta Health Services and the University of Alberta help families navigate the healthcare system and maintain regular dental visits [25]. These contextual supports may buffer the potential impact of maternal mental health and socio-digital pressures on dental care–seeking behaviours.

With respect to dietary behaviours, children’s sugar intake may be shaped more strongly by cultural food norms, food availability within households, and broader familial or community practices than by maternal mental health alone. Although maternal depression has been linked to poorer dietary routines in some studies [23,26], the absence of such associations in this sample may reflect specific cultural eating patterns or community supports within the immigrant populations studied [27]. These findings underscore the importance of situating parenting behaviours within culturally and digitally mediated contexts where social norms, acculturative processes, and socio-digital influences intersect.

A key finding of this study is the strong positive association between SMA and maternal mental health challenges. Mothers with higher levels of anxiety and depression reported significantly higher SMA, aligning with studies demonstrating that problematic social media use is closely tied to emotional vulnerability, social comparison, and coping processes [28,29]. For immigrant mothers, social media often functions as a socio-cultural lifeline for maintaining transnational relationships, engaging with ethnocultural communities, and accessing informal support networks. While these platforms can provide emotional support and a sense of connection, they may also intensify psychological strain through heightened exposure to social comparison, information overload, and persistent connectivity. This dual role may increase susceptibility to problematic or compulsive engagement with networked platforms, reflecting the broader socio-digital pressures that shape immigrant mothers’ emotional environments.

Despite a strong association, SMA did not mediate or moderate the relationship between maternal mental health and children’s OHBs. This suggests that social media addiction may function more as a marker of maternal psychological distress than as a direct behavioural mechanism influencing specific parenting practices such as children’s oral hygiene. Rather than shaping discrete caregiving behaviours, SMA appears to reflect the broader socio-digital ecology of immigrant motherhood, embedded within emotional, cultural, and technological contexts. This interpretation aligns with an emerging perspective in social media scholarship that considers social media not only as an individual behaviour but as embedded within socio-technical systems that shape family life, identity formation, and emotional well-being. For immigrant mothers, social media may both alleviate and exacerbate stress, offering vital social connections while simultaneously contributing to emotional overload, comparison pressures, and acculturative tension. Yet, these socio-digital forces may not uniformly influence all dimensions of parenting, affecting certain behaviours (such as emotional availability and consistency in routines) more than others (such as access to dental care).

Other contextual factors, such as maternal education level and length of residence in Canada, were associated with some oral health outcomes. Mothers with higher education and longer residence in Canada were more likely to report dental visits for their children, suggesting that acculturation and oral health literacy may buffer some of the negative effects of maternal distress on children’s oral health [30,31]. This emphasizes the importance of considering the interplay between digital assimilation, cultural adaptation, and parenting practices in immigrant families.

This study has notable strengths, including its focus on an understudied intersection of maternal mental health, SMA, and children’s oral health within a diverse immigrant population. Community-based recruitment through cultural brokers and multilingual supports enhanced inclusivity and representation of families typically underrepresented in health research. The use of validated measures (HADS and BSMAS) and assessment of multiple OHBs provided a comprehensive evaluation of maternal and child outcomes. The study also has limitations. The cross-sectional design precludes causal inference, and reliance on self-report data introduces potential for recall and social desirability bias. Although validated measures were used, their psychometric properties may vary across cultural and linguistic groups. Unmeasured factors, such as parental oral health literacy, digital literacy, parenting stress, and dental insurance, may have influenced outcomes. Future research should incorporate longitudinal designs and, critically, digital ethnography and platform-specific analyses to capture how social media practices are embedded in the everyday lives of immigrant families and how these evolving dynamics shape parenting behaviours over time. These findings highlight the complex interplay between maternal psychological well-being, social media use, and children’s oral health behaviours within immigrant families. By situating the study within the socio-digital context of immigrant parenting, this research contributes to a growing body of evidence calling for integrated, culturally grounded interventions that address both maternal mental health and digital media engagement.

## Conclusion

This study highlights an association between maternal mental health on children’s oral health behaviours within immigrant families. Higher levels of maternal anxiety and depression were associated with a lower likelihood of children brushing their teeth twice daily, with no significant associations observed for sugar intake or dental visits. Social media addiction was strongly linked to maternal psychological distress but did not mediate or moderate these relationships. Given the cross-sectional design, findings should be interpreted as associations rather than causal relationships. Overall, the results suggest that social media use reflects the broader socio-digital context of maternal well-being rather than directly influencing children’s oral health behaviours. Longitudinal and intervention-based studies are needed to clarify causal pathways and to assess whether integrating mental health and digital well-being into oral health promotion can support improved outcomes.

## Supporting information

S1 Data Deidendified data May 7, 2026. (XLSX)
